# Therapeutic Potential of *Holothuria leucospilota* Extract in STZ‐Induced Diabetic Rats: Targeting Inflammation and Apoptosis at the Gene Expression Level

**DOI:** 10.1002/edm2.70164

**Published:** 2026-01-18

**Authors:** Niloufar Darbandi, Muhanad Yusif Al‐rikabi

**Affiliations:** ^1^ Department of Biology, Faculty of Science Arak University Arak Iran

**Keywords:** diabetes mellitus, experimental, inflammation, sea cucumbers

## Abstract

**Introduction:**

Chronic inflammation is a key contributor to diabetes pathogenesis. Marine‐derived bioactive compounds offer a promising source of therapeutic agents. This study investigated the effects of *Holothuria leucospilota* n‐hexane extract on biochemical and inflammatory markers in diabetic male rats.

**Materials and Methods:**

Male Wistar rats were divided into four groups: control, diabetic, and diabetic treated with *H. leucospilota* extract (100 or 200 mg/kg). Diabetes was induced by streptozotocin. Animals received daily intraperitoneal injections of saline or extract. One‐third of the animals in each group (*n* = 8) were euthanised and sampled on days 1, 15, and 30. The extract composition was analysed by GC–MS. Serum glucose, insulin, amyloid beta, and expression of TGF‐β, TNF‐α, FasL, IL‐10, and miR‐146a were measured in blood, while leptin gene expression was assessed in liver samples.

**Results:**

GC–MS revealed major compounds including olean‐12‐ene‐3,28‐diol (14.1%), cyclohexane derivatives (8.2%), oleic acid (4.8%), and cis‐13‐eicosenoic acid (4.2%). Diabetic rats showed elevated glucose, amyloid beta, leptin, TGF‐β, TNF‐α, and FasL levels, with reduced insulin, IL‐10, and miR‐146a levels compared to the control group. Treatment with *H. leucospilota* extract (100 and 200 mg/kg) significantly reversed these changes at both 15 and 30 days compared to the diabetic group.

**Conclusion:**

*H. leucospilota* n‐hexane extract improved biochemical and inflammatory markers in diabetic rats, suggesting its potential as a natural therapeutic agent to mitigate diabetes‐associated inflammation and metabolic dysfunction. The identified bioactive compounds may underlie these beneficial effects, highlighting the therapeutic relevance of marine‐derived extracts in diabetes management.

## Graphical abstract

*Holothuria leucospilota* n‐hexane extract shows antidiabetic, anti‐inflammatory, and anti‐apoptotic effects in STZ‐induced diabetic rats. The extract modulates key pathways in glucose metabolism, insulin secretion, inflammation, and apoptosis. Treatment significantly decreased glucose and amyloid beta levels, increased insulin, and improved glycemic control and protection against amyloid toxicity. Expression of leptin, TGF‐β, TNF‐α, and FasL was downregulated, while IL‐10 and miR‐146a were upregulated, indicating reduced inflammation and enhanced anti‐inflammatory responses. These results support the potential of sea cucumber compounds as adjunct diabetes therapies; further clinical and toxicity studies are needed.

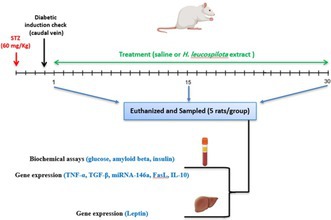

## 1 Introduction

Chronic metabolic and autoimmune disorders, particularly diabetes mellitus, represent a major and growing global health burden worldwide. The development of diabetes is influenced by multiple factors, including genetic predisposition, aging, obesity, and physical inactivity [1]. Type 2 diabetes mellitus (T2DM) is primarily characterised by insulin resistance accompanied by an inadequate compensatory insulin response, which arises from impairments in insulin signalling pathways and subsequent disturbances in glucose and lipid metabolism [2]. Among endocrine regulators, leptin—an adipose tissue–derived hormone—plays a pivotal role in the regulation of body weight, glucose homeostasis, and insulin secretion. Dysregulation of leptin signalling has been closely linked to obesity, systemic inflammation, and the pathogenesis of T2DM [3]. In addition to hormonal alterations, increasing evidence suggests that epigenetic regulators such as microRNAs (miRNAs) contribute to metabolic dysfunction in diabetes. Altered expression of miRNAs in pancreatic β‐cells and insulin target tissues has been reported under diabetic conditions, with miR‐146a emerging as a key mediator linking inflammation and metabolic regulation [4].

Chronic low‐grade inflammation is now recognised as a central mechanism underlying insulin resistance and the progression of diabetes‐related complications [1, 5]. In genetically susceptible individuals, age‐related weight gain is accompanied by sustained activation of inflammatory pathways and increased production of proinflammatory cytokines, particularly tumour necrosis factor‐α (TNF‐α) [6]. TNF‐α disrupts insulin signalling through activation of c‐Jun N‐terminal kinase (JNK) and nuclear factor‐κB (NF‐κB)–dependent pathways, thereby exacerbating insulin resistance and metabolic dysfunction. Conversely, anti‐inflammatory cytokines such as interleukin‐10 (IL‐10) exert protective effects by suppressing proinflammatory mediators and limiting excessive inflammatory responses, highlighting the importance of cytokine balance in metabolic homeostasis [7]. In parallel, transforming growth factor‐β (TGF‐β), a multifunctional cytokine involved in immune regulation, apoptosis, and tissue remodelling, has been implicated in diabetes pathophysiology. TGF‐β1 is expressed in pancreatic β‐cells and has been shown to influence β‐cell survival, while elevated circulating levels of TGF‐β1 have been reported in both type 1 and type 2 diabetes [8, 9]. Moreover, inflammatory conditions promote activation of apoptotic pathways, including Fas ligand (FasL) signalling, which contributes to accelerated β‐cell death and disease progression in diabetes [10].

Despite the availability of various pharmacological agents for diabetes management, effective strategies to prevent or attenuate long‐term hyperglycemic complications remain limited, and many current therapies are associated with adverse effects during prolonged use. Consequently, there is growing interest in identifying novel bioactive compounds with hypoglycemic, anti‐inflammatory, and antioxidant properties [11]. Marine organisms represent a rich source of biologically active natural products with potential metabolic benefits. Sea cucumbers, a group of echinoderms widely used in traditional medicine and functional foods, contain diverse bioactive constituents, including saponins, glycosaminoglycans, sulfated polysaccharides, and essential fatty acids [11, 12]. Experimental studies have demonstrated that sea cucumber–derived compounds, such as fucoidan and glycosaminoglycans, can improve hepatic inflammation, insulin resistance, and glucose metabolism in animal models of metabolic disorders [13]. Given the close interplay between inflammation, apoptosis, and insulin resistance in diabetes, the present study aimed to elucidate the endocrine‐ and inflammation‐related mechanisms underlying the metabolic effects of sea cucumber hexane extract by evaluating inflammatory and apoptotic gene expression in blood and liver tissue, along with serum biochemical parameters, in streptozotocin‐induced diabetic male rats.

### 1.1 Preparation and Extraction of Sea Cucumber Samples

Deep‐sea sea cucumber samples ( *Holothuria leucospilota* ) with an average weight of 380 g were collected by divers from the coast of Qeshm, Suza City, and transferred to the laboratory in large containers containing seawater with optimal aeration. The sea cucumber species ( *H. leucospilota* ) was identified and confirmed using the FAO (2012) identification key for sea cucumbers [14].

In sea cucumber samples, the body wall was separated from other parts by making an incision, divided into small pieces of 1–2 cm, and dried at 45°C until a constant weight was achieved. Approximately 50 g of dried sample was used for extraction. Dry samples were extracted using the Soxhlet method. To this aim, the n‐hexane solvent (Merck, Germany) in a ratio of 1:1.5 was added to Erlenmeyer flasks containing the dry sample and stored at 25°C for 72 h. The use of n‐hexane, a non‐polar solvent, was chosen to selectively extract lipophilic bioactive compounds (e.g., sterols and fatty acids) from sea cucumber, which are known for their anti‐diabetic, anti‐inflammatory, and antioxidant properties. Polar solvents mainly extract hydrophilic compounds, while n‐hexane also ensures compatibility with GC–MS analysis of non‐polar metabolites [15].

After filtering the samples, the solvent was removed using a rotary evaporator (Buchi Rota‐vapour R‐200, Switzerland), and samples were then purified at 45°C and 145 rpm [16]. The chemical composition of the hexane extract of sea cucumber was analysed using a Gas Chromatography–Mass Spectrometry (GC–MS) system (QP2010 SE, Japan). The analysis was performed using a DB‐5MS capillary column (30 m × 0.25 mm × 0.25 μm). Helium was used as the carrier gas at a constant flow rate of 1.2 mL/min. The oven temperature program was as follows: initially held at 50°C for 1 min, then ramped to 280°C at 8°C/min, followed by a further increase to 290°C at 20°C/min, with a final hold for 5 min. To analyse the volatile compounds in the extract, a headspace system was employed, in which the powdered hexane extract was heated at 90°C for 30 min prior to injection. The injector and detector temperatures were set according to manufacturer recommendations, and all analyses were carried out at the University of Tehran. This setup ensured optimal separation and detection of non‐polar bioactive compounds in the sea cucumber extract, compatible with GC–MS analysis.

### 1.2 Laboratory Animals, Grouping, and Treatment

A total of 96 male Wistar rats, approximately 40 days old and weighing 100–150 g, were purchased from the Razi Vaccine and Serum Research Institute in Tehran. The animals were kept in a 12‐h light–dark cycle with free access to food and water and were randomly divided into four groups (*n* = 24): control, diabetic, and diabetic + sea cucumber hexane extract (100, 200 mg/kg). The rats were made diabetic through STZ injection intraperitoneally (60 mg/kg). After 3 days, caudal vein sampling and blood sugar level measurement were performed using a glucometer (GlucoDr AGM, 2200, South Korea). An increase in blood sugar of > 300 mg/dL indicated a diabetic animal [17]. On the first day of the treatment phase, following diabetes induction, blood was collected from the heart and liver samples were taken from one‐third of the animals (*n* = 8) in each group. The remaining 16 animals in each group then entered the treatment phase. The control and diabetic groups received daily intraperitoneal injections of 0.5 cc saline, while the treated diabetic groups received daily intraperitoneal injections of *H. leucospilota* n‐hexane extract at doses of 100 or 200 mg/kg. In each group, half of the remaining animals (*n* = 8) were euthanised and sampled on day 15, and the other half (*n* = 8) on day 30 of treatment [18].

### 1.3 Preparation of Blood and Tissue Samples

In each group, the animals were anaesthetised with ether 12 h after the last injection, and blood was collected directly from the heart ventricle using a 2 mL syringe. Blood samples were centrifuged at 3000 rpm (Eppendorf, Germany) for 10 min, and the obtained serum was frozen at −80°C [19]. Serum samples were used to examine serum levels of glucose, amyloid beta, and insulin. Whole blood samples were used to examine the expression of TNF‐α, TGF‐β, miRNA‐146a, FasL, and IL‐10 genes. The leptin gene expression was examined using the liver tissue [20].

### 1.4 Serum Glucose, Amyloid Beta, and Insulin Levels

Serum glucose levels were measured by the enzymatic calorimetric method (Pars Azmoun kit, Iran) using an autoanalyser (Hitachi, Germany). Amyloid beta was quantified by ELISA using an East Biopharm research kit (China) with a sensitivity of 1.08 ng/mL and a coefficient of variation of 10%–12%. Serum insulin levels were measured by radioimmunoassay according to the kit instructions (Diagnostic DSL Systems Laboratories, USA).

### 1.5 RNA Extraction and cDNA Synthesis

RNA was extracted from blood cells and the liver tissue using an RNA extraction kit (Sambio, Taiwan) according to the manufacturer's protocol. The extracted RNA was stored at −70°C. The RNA concentration of each sample was measured with a NanoDrop spectrophotometer (Titertek Berthold, Germany) at the absorbance ratios of 260/280 and 280/230.

To synthesise cDNA with a kit (BIOFACT, Korea), 1000 ng of extracted RNA was mixed with Master Mix, Random Hexamer, and oligo dT and vortexed for a few seconds. The microtubes were incubated at 37°C for 5 min and then at 50°C for 30 min (for reverse transcription). Finally, the reaction mixture was treated at 95°C for 5 min (to inactivate the reverse transcriptase enzyme). The cDNA samples were stored at −20°C until PCR analysis.

### 1.6 Gene Expression Analysis

Gene expression levels were measured using qRT‐PCR on an ABI 7300 System (Applied Biosystems, USA). Each 20 μL reaction contained 10 μL BIOFACT Mastermix, 2 μL cDNA, 1 μL forward primer, 1 μL reverse primer, and 6 μL nuclease‐free water. Initial denaturation was performed at 95°C for 15 min, followed by 40 cycles of denaturation at 95°C for 20 s, and annealing/elongation at 60°C for 40 s. Melting curves were generated from 60°C to 95°C to confirm primer specificity. Gene expression was quantified relative to the internal reference gene GAPDH using the 2^−ΔΔCT^ method. Primers were designed and synthesised by KavoshGene Company (Table 1), and their specificity was confirmed by BLAST and melting curve analysis.

**TABLE 1:** Sequences of primers used in Real‐time PCR.

Gene	Primer direction	Primer sequence (5′ → 3′)	Length (bp)
*GAPDH*	Forward	AGACAGCCGCATCTTCTTGT	20
	Reverse	CTTGCCGTGGGTAGAGTCAT	20
*LEP*	Forward	AGGTGGAGGTGAACTGGACGG	21
	Reverse	GGCCCACAAAGTCCTCTCAGCAC	23
*TGF‐β*	Forward	CCCAGCATCTGCAAAGCTC	19
	Reverse	GTCAATGTACAGCTGCCGCA	20
*IL‐10*	Forward	GCTCAGCACTGCTATGTTGC	20
	Reverse	TTCATGGCCTTGTAGACACC	20
*TNF‐α*	Forward	CTGAGGAGAGCCCATTTGAG	20
	Reverse	GGTGTGCACAGAGAGGAGAG	20
*FasL*	Forward	CATGCAGCAGCCCATGAATTAC	22
	Reverse	CTCTAGGCCCACAAGATGGACAG	23
*miR‐146a*	Forward	CCGCGCTGAGAACTGAATTCCA	22
	Reverse	AGTGCAGGGTCCGAGGTATT	20

### 1.7 Statistical Analysis

Data were statistically analysed with the one‐way analysis of variance (ANOVA) using SPSS software. Results were reported as mean ± SEM at a significance level of *p* < 0.05. All measurements were performed in biological triplicates.

## 2 Results

### 2.1 Results of GC‐MASS Analysis of *H. leucospilota* Hexane Extract

The GC–MS analysis revealed that the extract contained a variety of terpene‐terpenoid compounds, including olean‐12‐ene‐3,28‐diol (3β) (14.116%) and cyclohexane, 1,1‐bis(5‐methyl‐2‐furyl) (8.225%), as well as fatty acids such as oleic acid (4.753%) and cis‐13‐eicosenoic acid (4.228%), which were among the major constituents of the extract (Table 2). The percentages are based on GC–MS peak areas, and only the major compounds are reported.

**TABLE 2:** The main components identified in the n‐hexane extract of the body wall of *H. leucospilota*.

Category	Compounds	Retention time	Area %
Terpenes and terpenoid	Olean‐12‐ene‐3,28‐diol, (3β)	10.232	14.116
	Cyclohexane, 1,1‐bis(5‐methyl‐2‐furyl)	11.749	8.225
	Phorbol	12.224	6.228
	Lumisantonin	11.071	6.130
	Neoisolongifolene, 8‐bromo	10.893	5.992
Fatty acids	Oleic Acid	14.68	4.753
	cis‐13‐Eicosenoic acid	19.91	4.228
	Arachidonic acid	19.84	2.776
	Palmitoleic acid	17.929	2.065
	Eicosanoic acid	20.9	1.560
	22‐Tricosenoic acid	25.379	1.540
Others	Tetracyclo [6.1.0.0(2,4)0.0(5,7)] nonane, 3,3,6,6,9,9‐hexaethyl‐, cis, cis, trans—	11.241	8.402
	1‐Heptatriacotanol	12.434	5.035
	1,4‐Hexadien‐3‐one, 5‐methyl‐1‐[2,6,6‐trimethyl‐2,4‐cyclohexadien‐1‐yl]—	12.907	4.114
	Propanoic acid‐3‐oxo, 3‐(2,4‐dichlorophenyl)‐, ethyl ester	10.380	3.721
	Hexadecanoic acid, 2‐hydroxy‐1‐(hydroxymethyl)ethyl ester	23.632	2.694
	17‐Pentatriacontene	24.919	2.242

### 2.2 Serum Levels of Insulin, Glucose, and Amyloid Beta in the Experimental Groups

The results of one‐way ANOVA showed statistically significant differences in the mean variables of blood glucose, insulin, and amyloid beta between the experimental groups on days 1, 15, and 30 (*p* < 0.001) (Table 3). The serum levels of glucose and amyloid beta increased significantly, and the insulin level decreased significantly in the groups receiving STZ compared to the control group. The serum levels of glucose and amyloid beta decreased significantly, and the insulin level increased in diabetic rats treated with *H. leucospilota* n‐hexane extract (100 and 200 mg/kg) on days 15 and 30 of treatment compared to the diabetic group.

**TABLE 3:** Comparison of mean serum glucose, insulin hormone, and amyloid beta protein levels on days 1, 15, and 30 in the experimental groups.

Groups					
Factors	Days	Control	Diabetic	Diabetic + extract (100 mg/kg)	Diabetic + extract (200 mg/kg)
Blood glucose (mg/dL)	1	93.1 ± 11.1^b^	421.6 ± 8.5^a^	440.9 ± 13.5^a^	438.6 ± 14.5^a^
	15	107.6 ± 8.8^d^	431.5 ± 14.6^a^	259.2 ± 10.5^b^	184.6 ± 5.0^c^
	30	94.3 ± 4.3^d^	508.5 ± 11.2^a^	233.6 ± 12.0^b^	159.5 ± 7.5^c^
Insulin (micU/mL)	1	13.8 ± 2.6^a^	5.8 ± 1.4^b^	5.7 ± 1.3^b^	5.3 ± 1.7^b^
	15	12.5 ± 1^a^	5.5 ± 1.3^c^	6.4 ± 2.1^bc^	7.9 ± 2.0^b^
	30	13.5 ± 1.8^a^	4.3 ± 0.8^c^	7.4 ± 1.1^b^	8.6 ± 2.6^b^
Amyloid beta (nmol/mg)	1	0.133 ± 0.01^b^	0.359 ± 0.01^a^	0.328 ± 0.02^a^	0.318 ± 0.03^a^
	15	0.114 ± 0.02^d^	0.303 ± 0.01^a^	0.157 ± 0.03^c^	0.204 ± 0.04^b^
	30	0.094 ± 0.01^d^	0.381 ± 0.02^a^	0.183 ± 0.04^c^	0.224 ± 0.02^b^

*Note:* All values are given as Mean ± SEM. The values with different superscript letters within the same row significantly differ at *p* < 0.05.

### 2.3 Quantitative Analysis of Changes in Leptin, miR‐146a, TGF‐β, IL‐10, TNF‐α, and FasL Gene Expression Levels Using Real‐Time PCR

The effect of *H. leucospilota* n‐hexane extract on the expression of the target genes in diabetic rats was evaluated using real‐time PCR analysis. After confirming the specificity of the bands observed in semi‐quantitative RT‐PCR, the expression of the studied genes was quantified with the PCR technique (35 reaction cycles).

Analysis of melting and amplification curves demonstrated sharp single peaks for leptin, miR‐146a, TGF‐β, IL‐10, TNF‐α, and FasL PCR products, confirming the specificity of amplification. No primer‐dimer artefacts were observed, and negative control samples showed no detectable amplification.

**FIGURE 1:**
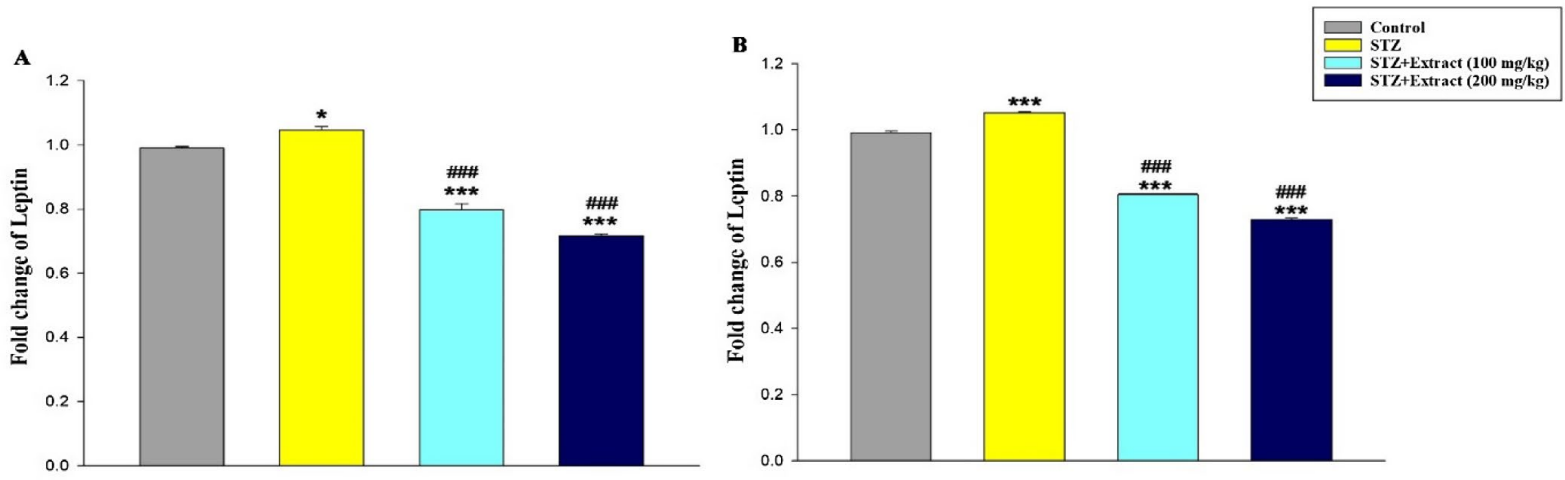
Expression of leptin gene over a period of 15 (A) and 30 (B) days in different experimental groups using Real‐time‐PCR technique. Data are presented as mean ± SEM of eight animals per group. *p < 0.05, ***p < 0.001 as compared with control group. ^###^ *p* < 0.001 as compared with STZ group.

**FIGURE 2:**
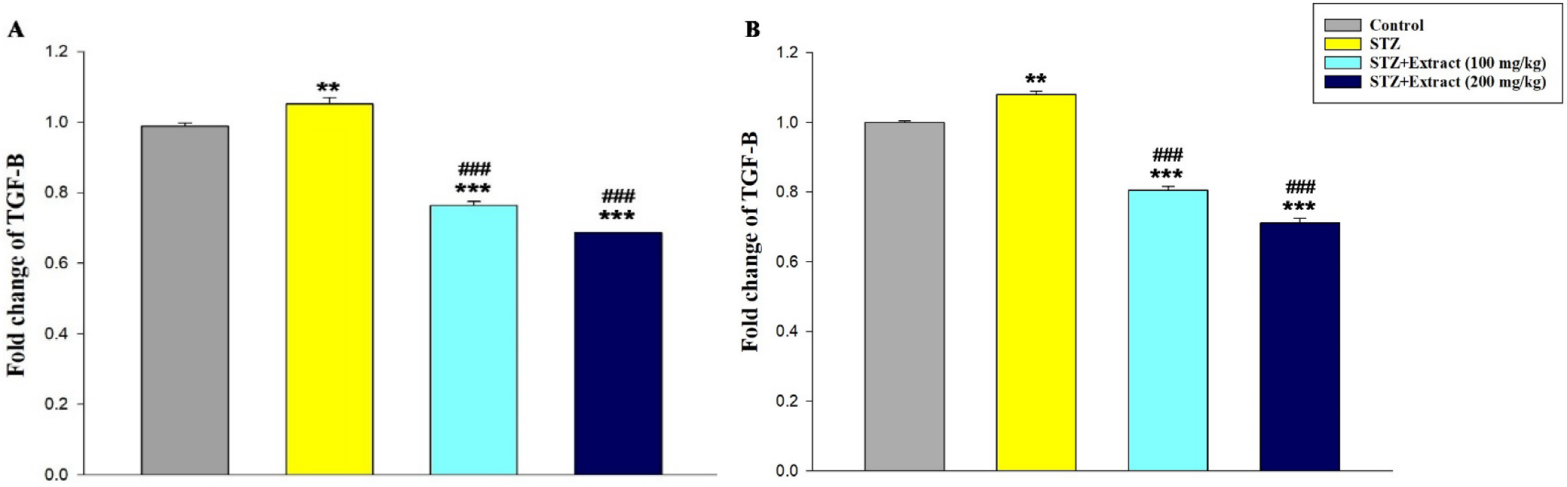
Expression of TGF‐β gene over a period of 15 (A) and 30 (B) days in different experimental groups using Real‐time‐PCR technique. Data are presented as mean ± SEM of eight animals per group. **p < 0.01, ***p < 0.001 as compared with control group. ^**###**^ *p* < 0.001 as compared with STZ group.

**FIGURE 3:**
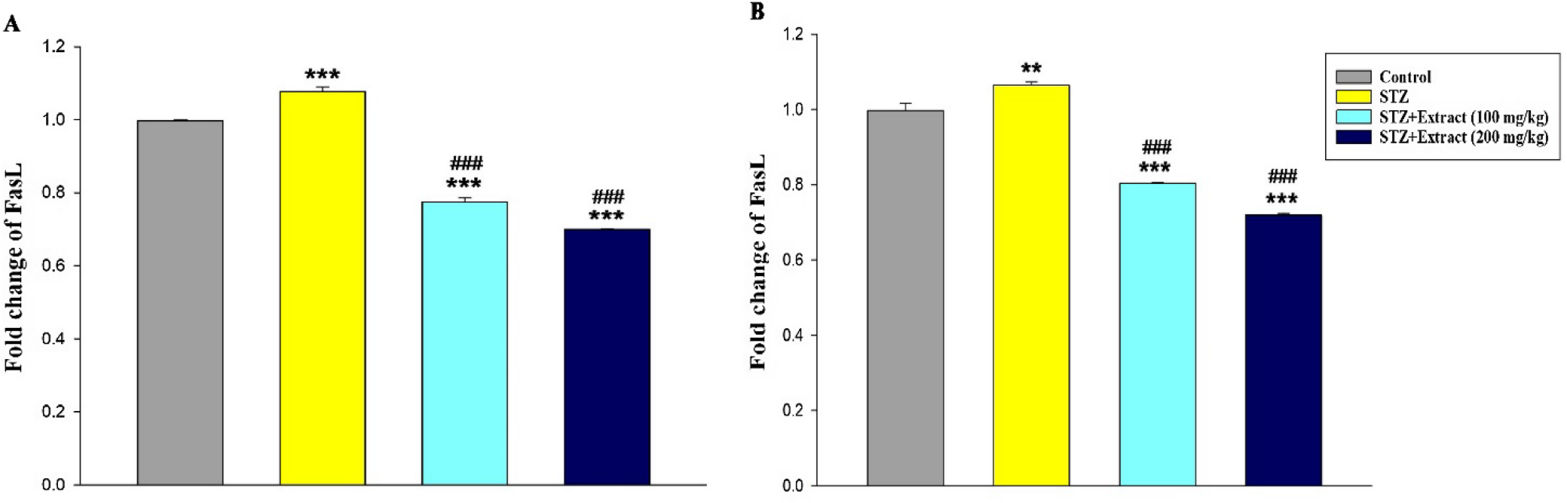
Expression of FasL gene over a period of 15 (A) and 30 (B) days in different experimental groups using Real‐time‐PCR technique. Data are presented as mean ± SEM of eight animals per group. **p < 0.01, ***p < 0.001 as compared with control group. ^**###**^ *p* < 0.001 as compared with STZ group.

**FIGURE 4:**
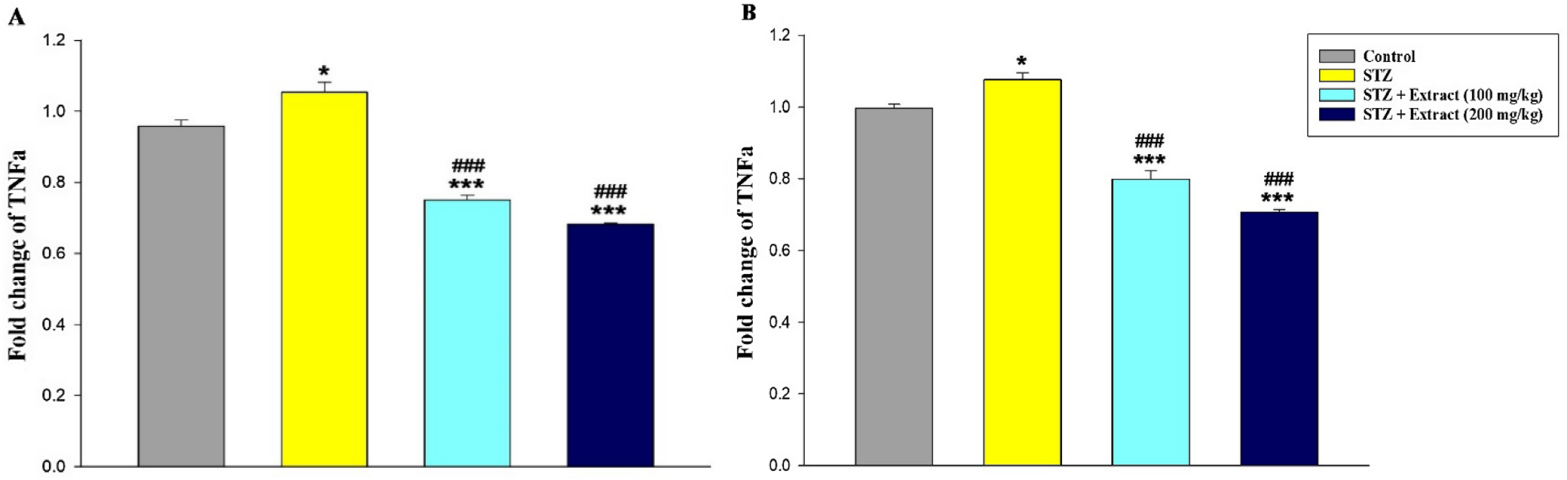
Expression of TNF‐α gene over a period of 15 (A) and 30 (B) days in different experimental groups using Real‐time‐PCR technique. Data are presented as mean ± SEM of eight animals per group. *p < 0.05, ***p < 0.001 as compared with control group. ^**###**^ *p* < 0.001 as compared with STZ group.

Data analysis indicated that leptin gene expression (Figure 1A,B) in the liver tissue and TGF‐β (Figure 2A,B), FasL (Figure 3A,B), and TNF‐α (Figure 4A,B) genes increased significantly in the blood of diabetic rats in 15‐day and 30‐day periods compared to the control group. According to the results, significant decreases in the expression of these genes were observed in diabetic rats treated with *H. leucospilota* hexane extract at concentrations of 100 and 200 mg/kg in both periods of 15 and 30 days compared to the diabetic group.

**FIGURE 5:**
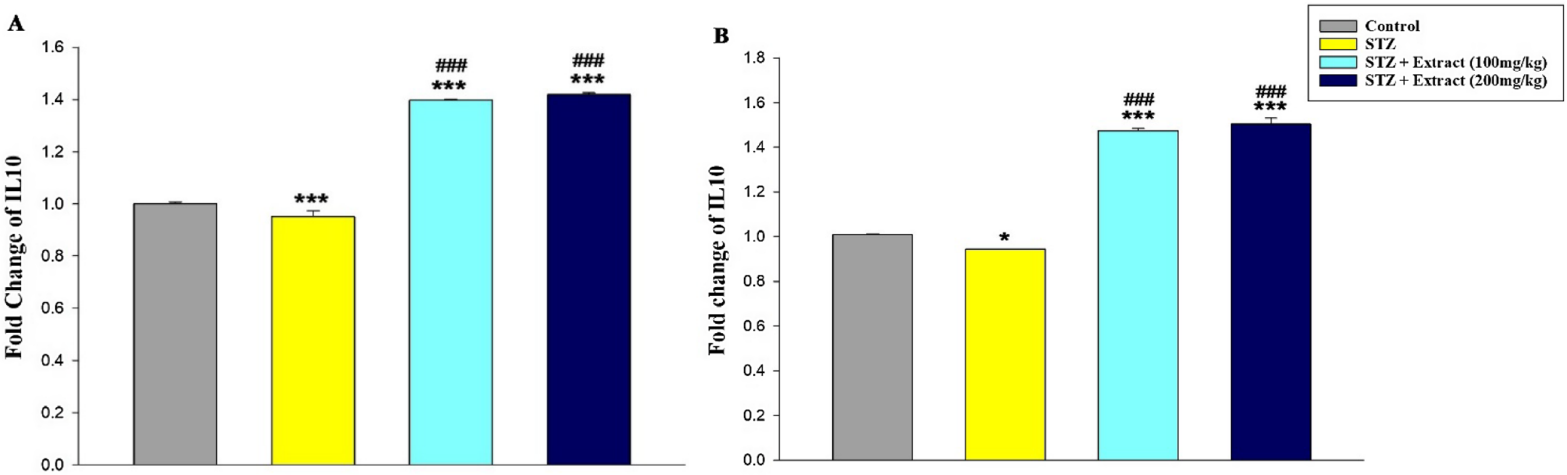
Expression of IL‐10 gene over a period of 15 (A) and 30 (B) days in different experimental groups using Real‐time‐PCR technique. Data are presented as mean ± SEM of eight animals per group. *p < 0.05, ***p < 0.001 as compared with control group. ^**###**^ *p* < 0.001 as compared with STZ group.

**FIGURE 6:**
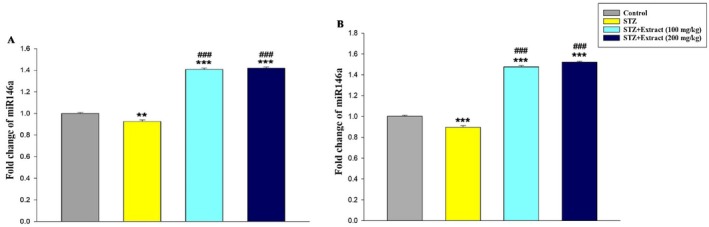
Expression of miR‐146a gene over a period of 15 (A) and 30 (B) days in different experimental groups using Real‐time‐PCR technique. Data are presented as mean ± SEM of eight animals per group. **p < 0.01, ***p < 0.001 as compared with control group. ^**###**^ *p* < 0.001 as compared with STZ group.

The analyses also showed that the expression of IL‐10 (Figure 5A,B) and miR‐146a (Figure 6A,B) decreased significantly in the blood of diabetic rats in the periods of 15 and 30 days compared to the control group. In addition, a significant increase in the expression of these genes was seen in diabetic rats treated with *H. leucospilota* hexane extract at concentrations of 100 and 200 mg/kg in both periods of 15 and 30 days compared to the diabetic group.

## 3 Discussion

STZ enters pancreatic beta cells via GLUT2 and causes DNA alkylation and damage. This damage activates the poly ADP ribosylation repair process, which destructs pancreatic tissue by producing free radicals, leading to hyperglycemia [21]. The resulting increase in islet amyloid polypeptide (amylin), produced and secreted along with insulin by pancreatic β cells, promotes the formation of toxic amyloid oligomers. These oligomers contribute to the development of diabetes, apoptosis, and progressive beta cell failure by activating the extrinsic apoptosis pathway through reactive oxygen species generation, impaired mitochondrial function, chromatin condensation, and other apoptotic mechanisms [22, 23].

Sea cucumber extract contains antioxidants and bioactive compounds, including flavonoids, saponins, alkaloids, eicosapentaenoic acid, and docosahexaenoic acid, which have been reported to exert antidiabetic effects [24]. The fatty acids present in the extract may enhance tissue insulin sensitivity by inhibiting alpha‐glucosidase activity [25, 26], while saponins contribute to the reduction of inflammation and improvement of insulin resistance [27]. Fucoidans derived from sea cucumber can promote glucose transport in skeletal muscle and increase insulin sensitivity [28], and glycosaminoglycans may suppress hepatic glucose production [29]. Collectively, these mechanisms are expected to support better glycemic control and modulate factors involved in β‐cell dysfunction [30].

Amplified leptin levels are associated with the risk of diabetes or obesity. Leptin production by white adipose tissue increases significantly in diabetic individuals and leads to reduced glucose tolerance by lowering insulin sensitivity. Elevated leptin levels result in declined responsiveness of pancreatic β cells, inability to suppress insulin secretion, and hyperinsulinemia. This can in turn exacerbate obesity, increase leptin gene expression, and amplify its level in blood serum. In diabetic individuals, therefore, increased leptin expression in a positive feedback loop causes metabolic disorders [31].

Bioactive metabolites present in sea cucumber extract reduce the transcription of appetitive neuropeptides and regulate glucose homeostasis by reducing endoplasmic reticulum stress and phosphorylation of JAK2/STAT3 pathway proteins. On the other hand, they reduce leptin secretion by increasing the levels of stress hormones (catecholamines and cortisol) and inhibiting the phosphodiesterase nucleotide cycle [32].

TGF‐β plays a pivotal role in the development of insulin resistance and obesity. Plasma levels of TGF‐β1 rise significantly in diabetic patients. Elevated blood sugar levels increase the TGF‐β1 gene transcription by increasing protein kinase C (PKC) enzyme activity [11]. Long‐term and chronic inflammation caused by metabolic disorders (e.g., diabetes) increases TGF‐β expression by inducing oxidative stress, leading to excessive deposition of collagen fibres in the extracellular matrix and fibrosis formation. Bioactive metabolites of sea cucumber extract can inhibit tissue fibrosis and reduce TGF‐β gene expression by suppressing the TGF‐β/SMAD pathway [33]. Treatment with eicosapentaenoic acid contained in sea cucumber extract significantly inhibits the activation of TGF‐β and Smad3, increases the phosphorylation level of PI3K/AKT, suppresses NF‐κB activation, reduces the expression of proinflammatory cytokines, and suppresses oxidative stress and the mitochondrial‐mediated apoptosis signalling pathway [34].

FasL seems to be the likely mediator for the lysis of Islets of Langerhans. The level and timing of Fas expression by Islets of Langerhans is induced by incubation with IL‐1β and IFNɣ. A significant increase in Fas gene expression occurs in an inflammatory environment and in response to cytokines, which is accompanied by an accelerated phase of beta cell death [13]. Saponins and fucoidans in sea cucumber extract seem to reduce FasL expression by inhibiting NF‐kB activity in T cells [35].

TNF‐α appears to play a direct role in the development and progression of diabetic complications through cell apoptosis and increased expression of inflammatory factors [36] and inhibits insulin receptor signalling and activity by reducing GLUTA4 [37]. Conversely, IL‐10 has profound and diverse anti‐inflammatory effects; this cytokine inhibits the expression of inflammatory interleukins [7]. In vitro studies have documented that IL‐10 protects against TNF‐α‐dependent insulin resistance in adipocytes [8]. By increasing IL‐10 levels, sea cucumber extract inhibits hepatic inflammation in diabetic rats and improves insulin systemic function [38].

Dysregulation of miRNA expression affects a variety of important cellular functions and significantly impacts health and diabetes development. miR‐146a prevents the increased production of inflammatory cytokines in diabetic patients and restores β‐cell function in T2DM through the NUMB/β‐catenin signalling pathway [39]. High blood sugar levels in diabetic individuals cause a decrease in miR‐146a expression [40]. Fucoidans, polysaccharides found in sea cucumber, have potential useful hypoglycemic effects in diabetes treatment; these compounds can increase miRNA‐29 and miRNA‐146a levels [41]. Saponins also regulate the IRAK1/TRAF6 pathway by increasing the expression of miR‐146a, reducing inflammation, and improving diabetes by inhibiting the NF‐κB pathway [42].

## 4 Limitations

Although the STZ‐induced diabetes model is widely used, it replicates only certain features of diabetes, and direct extrapolation of the results to humans is limited. The sample size (*n* = 8 per group) may also restrict the statistical power to detect subtle differences, indicating the need for studies with larger sample sizes. Moreover, in this study, the crude n‐hexane extract of Hikomaruta was used, and it would be preferable in future research to evaluate the effects of individual active compounds separately. Additionally, future investigations should assess the toxicity of the extract and its long‐term effects on diabetes, inflammation, and related molecular pathways.

## 5 Conclusion

This study highlights the therapeutic potential of *H. leucospilota* n‐hexane extract as a multifunctional modulator of diabetes‐related metabolic dysfunction. The findings emphasise that the beneficial effects of the extract extend beyond glycemic regulation and involve coordinated regulation of inflammatory, apoptotic, and endocrine‐related molecular pathways. By targeting key mediators that link chronic inflammation, β‐cell dysfunction, and insulin resistance, sea cucumber–derived bioactive compounds may contribute to preserving metabolic homeostasis in diabetes. From a scientific perspective, this work underscores the relevance of marine natural products as sources of bioactive molecules capable of influencing epigenetic and immune‐related mechanisms implicated in diabetes progression. These insights provide a mechanistic framework for future studies aimed at isolating active compounds and exploring their translational potential as adjunct strategies in diabetes management.

## Author Contributions

N.D. and M.Y.A.: designed the experiments; N.D.: Supervised, directed and managed the study; M.Y.A.: performed experiments and collected data. N.D.: prepared figures and tables, wrote the main manuscript text; N.D. and M.Y.A.: discussed the results and strategy and final approved of the version to be published.

## Funding

The authors have nothing to report.

## Ethics Statement

All procedures were approved by the local ethical committee (Research and Ethics Committee of the School of Biology, University of Arak; IR.ARAKMU.REC.1403.039) and were carried out in accordance with the ethical standards and principles of laboratory animal care (NIH publication) and animal protection laws.

Animal Studies: All animal experiments complied with relevant guidelines and regulations.

## Consent

The authors have nothing to report.

## Conflicts of Interest

The authors declare no conflicts of interest.

## Data Availability

The datasets used and/or analysed during the current study are available from the corresponding author on reasonable request.

## References

[1] M. Lotfy , J. Adeghate , H. Kalasz , J. Singh , and E. Adeghate , “Chronic Complications of Diabetes Mellitus: A Mini Review,” Current Diabetes Reviews 13 (2017): 3–10, 10.2174/1573399812666151016101622. 26472574

[2] E. Ahmad , S. Lim , R. Lamptey , D. R. Webb , and M. J. Davies , “Type 2 Diabetes,” Lancet 400 (2022): 1803–1820, 10.1016/S0140-6736(22)01655-5. 36332637

[3] K. Rehman , M. S. H. Akash , and Z. Alina , “Leptin: A New Therapeutic Target for Treatment of Diabetes Mellitus,” Journal of Cellular Biochemistry 119 (2018): 5016–5027. 29236298 10.1002/jcb.26580

[4] E. Roggli , S. Gattesco , D. Caille , et al., “Changes in microRNA Expression Contribute to Pancreatic β‐Cell Dysfunction in Prediabetic NOD Mice,” Diabetes 61 (2012): 1742–1751. 22537941 10.2337/db11-1086 PMC3379668

[5] B. Shamshadi , R. Askari , R. Rezaei , and A. H. Haghighi , “Investigation of an Increase in the Expression of the Mir146a Gene in the Hippocampus and a Decrease in the Levels of Blood Sugar, Insulin, and Insulin Resistance in Elderly Diabetic Rats After a Period of High Intensity Interval Training,” Diabetes, Metabolic Syndrome and Obesity: Targets and Therapy 14 (2023): 75–86.

[6] M. Satoh , M. Nakamura , H. Satoh , H. Saitoh , I. Segawa , and K. Hiramori , “Expression of Tumor Necrosis Factor‐Alpha–Converting Enzyme and Tumor Necrosis Factor‐Alpha in Human Myocarditis,” Journal of the American College of Cardiology 36 (2000): 1288–1294. 11028485 10.1016/s0735-1097(00)00827-5

[7] P. Feng , J. Chai , M. Zhou , N. Simon , L. Huang , and H. Wang , “Interleukin‐10 Is Produced by a Specific Subset of Taste Receptor Cells and Critical for Maintaining Structural Integrity of Mouse Taste Buds,” Journal of Neuroscience 34 (2014): 2689–2701. 24523558 10.1523/JNEUROSCI.3074-13.2014 PMC3921433

[8] X. Yuan , X. Dai , L. Liu , et al., “Comparing the Effects of 6 Months Aerobic Exercise and Resistance Training on Metabolic Control and β‐Cell Function in Chinese Patients With Prediabetes: A Multicenter Randomized Controlled Trial,” Journal of Diabetes 12 (2020): 25–37. 31141300 10.1111/1753-0407.12955

[9] S. Xie , P. Macedo , M. Hew , C. Nassenstein , K.‐Y. Lee , and K. F. Chung , “Expression of Transforming Growth Factor‐β (TGF‐β) in Chronic Idiopathic Cough,” Respiratory Research 10 (2009): 1–10. 19463161 10.1186/1465-9921-10-40 PMC2688489

[10] F. Heydarpour , S. Sajadimajd , E. Mirzarazi , et al., “Involvement of TGF‐β and Autophagy Pathways in Pathogenesis of Diabetes: A Comprehensive Review on Biological and Pharmacological Insights,” Frontiers in Pharmacology 11 (2020): 498758. 33041786 10.3389/fphar.2020.498758 PMC7522371

[11] J. Kelley , “Transformin Growth Factor‐β,” in Cytokines of the Lung (CRC Press, 2022), 101–137.

[12] S. F. Gallagher , J. Yang , K. Baksh , et al., “Acute Pancreatitis Induces FasL Gene Expression and Apoptosis in the Liver,” Journal of Surgical Research 122 (2004): 201–209. 15555619 10.1016/j.jss.2004.05.019

[13] M. Pearl‐Yafe , E. S. Yolcu , I. Yaniv , J. Stein , H. Shirwan , and N. Askenasy , “The Dual Role of Fas‐Ligand as an Injury Effector and Defense Strategy in Diabetes and Islet Transplantation,” BioEssays: News and Reviews in Molecular, Cellular and Developmental Biology 28 (2006): 211–222. 16435302 10.1002/bies.20356

[14] S. Al Azad , S. R. M. Shaleh , and S. Siddiquee , “Comparison of Fatty Acid and Proximate Composition Between *Holothuria edulis* and *Holothuria scabra* Collected From Coastal Water of Sabah, Malaysia,” Advances in Bioscience and Biotechnology 8 (2017): 91–103.

[15] R. K. Saini , P. Prasad , X. Shang , and Y.‐S. Keum , “Advances in Lipid Extraction Methods—A Review,” International Journal of Molecular Sciences 22 (2021): 13643. 34948437 10.3390/ijms222413643 PMC8704327

[16] B. Farjami , M. A. Nematollahi , Y. Noradi , G. Irajian , and M. Nazemi , “Study of Antibacterial Effect of the Extracts of the Sea Cucumber (*Holothuria leucospilota*) of Persian Gulf on the *Escherichia coli* ,” Iranian Journal of Medical Microbiology 8 (2014): 27–33.

[17] S. Romina and M. Norozi , “The Effect of Hydroalcoholic Extract of *Nasturtium officinale* on Blood Glucose of Diabetic Mice,” Journal of Islamic and Iranian Traditional Medicine 8 (2017): 315–321.

[18] F. Najafi , N. Goodarzi , M. M. Zangeneh , A. Zangeneh , and L. Hagh‐Nazari , “Antidiabetic and Hepatoprotective Effects of Bitter Fraction of *Stevia rebaudiana* Alcoholic Extract on Streptozotocin‐Induced Diabetic Male Mice,” Journal of Rafsanjan University of Medical Sciences 16 (2017): 493–504.

[19] S. D. Sadoughi and M. Chamipa , “Effects of Aqueous Extract of *Holothuria arenicola* and Low Frequency Electromagnetic Field on Serum Insulin, Glucose and Beta‐Amyloid (Aβ1‐42) in Diabetic Rats,” Feyz Journal of Medical Sciences 20 (2016): 1–10.

[20] G. Frühbeck , V. Catalán , A. Rodríguez , et al., “Normalization of Adiponectin Concentrations by Leptin Replacement in Ob/Ob Mice Is Accompanied by Reductions in Systemic Oxidative Stress and Inflammation,” Scientific Reports 7 (2017): 2752. 28584304 10.1038/s41598-017-02848-0 PMC5459809

[21] E. G. Maxim , O. K. Lunin , T. V. Sharapov , and S. B. Parfenyuk , “Peroxyredoxin 6 Protects RIN‐M5F Pancreatic Beta Cells Against Streptozotocin‐Induced Senescence,” Cellular Physiology and Biochemistry 58 (2024): 527–537. 39348523 10.33594/000000729

[22] E. Leyva‐García , R. Lara‐Martínez , L. Morán‐Zanabria , et al., “Novel Insight Into Streptozotocin‐Induced Diabetic Rats From the Protein Misfolding Perspective,” Scientific Reports 7 (2017): 11552. 28912603 10.1038/s41598-017-11776-y PMC5599686

[23] V. Moya‐Gudiño , N. F. Altamirano‐Bustamante , C. Revilla‐Monsalve , and M. M. Altamirano‐Bustamante , “Decoding the Contribution of IAPP Amyloid Aggregation to Beta Cell Dysfunction: A Systematic Review and Epistemic Meta‐Analysis of Type 1 Diabetes,” International Journal of Molecular Sciences 26 (2025): 767. 39859479 10.3390/ijms26020767 PMC11766435

[24] P. Chandika , P. Tennakoon , T.‐H. Kim , et al., “Marine Biological Macromolecules and Chemically Modified Macromolecules; Potential Anticoagulants,” Marine Drugs 20 (2022): 654. 36286477 10.3390/md20100654 PMC9604568

[25] T. L. Wargasetia , and N. Widodo , “Mechanisms of Cancer Cell Killing by Sea Cucumber‐Derived Compounds,” Investigational New Drugs 35 (2017): 820–826. 28920157 10.1007/s10637-017-0505-5 PMC5694523

[26] M. A. Zarei and H. Tahazadeh , “Alpha‐Glucosidase Inhibitory Activity in Methanol Extract of Some Plants From Kurdistan Province,” Journal of Medicinal Plants 18 (2019): 227–235.

[27] P. Lin , N. Shen , F. Yin , and S. D. Guo , “Sea Cucumber‐Derived Compounds for Treatment of Dyslipidemia: A Review,” Frontiers in Pharmacology 13 (2022): 1000315. 36188620 10.3389/fphar.2022.1000315 PMC9515789

[28] J. Wang , S. Hu , W. Jiang , W. Song , L. Cai , and J. Wang , “Fucoidan From Sea Cucumber May Improve Hepatic Inflammatory Response and Insulin Resistance in Mice,” International Immunopharmacology 31 (2016): 15–23. 26690975 10.1016/j.intimp.2015.12.009

[29] Y. Chen , Y. Wang , S. Yang , M. Yu , T. Jiang , and Z. Lv , “Glycosaminoglycan From *Apostichopus japonicus* Improves Glucose Metabolism in the Liver of Insulin Resistant Mice,” Marine Drugs 18 (2019): 1. 31861309 10.3390/md18010001 PMC7024160

[30] N. Nicosia , I. Kwiecień , M. Bednarski , et al., “Anti‐Diabetes and Neuroprotection Potential and Primary Safety Studies of *Isatis tinctoria L*. Hydroalcoholic Leaf Extract,” Fitoterapia 177 (2024): 106138. 39053741 10.1016/j.fitote.2024.106138

[31] Y. Hernández‐Díaz , M. de los Ángeles Ovando‐Almeida , A. Fresán , et al., “Increased Leptin Levels in Plasma and Serum in Patients With Metabolic Disorders: A Systematic Review and Meta‐Analysis,” International Journal of Molecular Sciences 25 (2024): 12668. 39684379 10.3390/ijms252312668 PMC11641391

[32] B. Jamali Gharakhanlou , A. Ameghani , and A. Zarghami Khameneh , “Effects of Simultaneous Interventions of Caffeine and Aerobic Training on Leptin‐to‐Adiponectin Ratio (LAR) in Type 2 Diabetic Men,” Iranian Journal of Diabetes and Metabolism 21 (2021): 139–150.

[33] Z. Wang , R. Xu , H. Yang , et al., “Vitamin E Regulates the Collagen Contents in the Body Wall of Sea Cucumber (*Apostichopus japonicus*) via Its Antioxidant Effects and the TGF‐β/Smads Pathway,” Antioxidants 13 (2024): 847. 39061914 10.3390/antiox13070847 PMC11274103

[34] H. H. Shi , L.‐Y. Zhang , L.‐P. Chen , et al., “EPA‐Enriched Phospholipids Alleviate Renal Interstitial Fibrosis in Spontaneously Hypertensive Rats by Regulating TGF‐β Signaling Pathways,” Marine Drugs 20 (2022): 152. 35200681 10.3390/md20020152 PMC8879699

[35] J. L. di Gesso , J. S. Kerr , Q. Zhang , et al., “Flavonoid Metabolites Reduce Tumor Necrosis Factor‐α Secretion to a Greater Extent Than Their Precursor Compounds in Human THP‐1 Monocytes,” Molecular Nutrition & Food Research 59 (2015): 1143–1154. 25801720 10.1002/mnfr.201400799 PMC4973837

[36] H. Bashir , S. Ahmad Bhat , S. Majid , et al., “Role of Inflammatory Mediators (TNF‐α, IL‐6, CRP), Biochemical and Hematological Parameters in Type 2 Diabetes Mellitus Patients of Kashmir, India,” Medical Journal of the Islamic Republic of Iran 34 (2020): 5. 32284929 10.34171/mjiri.34.5 PMC7139256

[37] M. Guo and X. Wang , “Pathological Mechanism and Targeted Drugs of Ulcerative Colitis: A Review,” Medicine (Baltimore) 102 (2023): e35020. 37713856 10.1097/MD.0000000000035020 PMC10508406

[38] H. Tutunchi , A. Ostadrahimi , M. Saghafi‐Asl , et al., “Expression of NF‐κB, IL‐6, and IL‐10 Genes, Body Composition, and Hepatic Fibrosis in Obese Patients With NAFLD—Combined Effects of Oleoylethanolamide Supplementation and Calorie Restriction: A Triple‐Blind Randomized Controlled Clinical Trial,” Journal of Cellular Physiology 236 (2021): 417–426. 32572955 10.1002/jcp.29870

[39] B. Alipoor , H. Ghaedi , R. Meshkani , et al., “Association of MiR‐146a Expression and Type 2 Diabetes Mellitus: A Meta‐Analysis,” International Journal of Molecular and Cellular Medicine 6 (2017): 156. 29682487 10.22088/acadpub.BUMS.6.3.156 PMC5898639

[40] M. N. Poy , L. Eliasson , J. Krutzfeldt , et al., “A Pancreatic Islet‐Specific microRNA Regulates Insulin Secretion,” Nature 432 (2004): 226–230. 15538371 10.1038/nature03076

[41] C. Zhao , S. Lai , D. Wu , et al., “miRNAs as Regulators of Antidiabetic Effects of Fucoidans,” eFood 1 (2020): 2–11.

[42] Y. Du , W. Chen , Y. Li , D. Liang , and G. Liu , “Study on the Regulatory Effect of Panax Notoginseng Saponins Combined With Bone Mesenchymal Stem Cell Transplantation on IRAK1/TRAF6‐NF‐κB Pathway in Patients With Diabetic Cutaneous Ulcers,” Journal of Orthopaedic Surgery and Research 18 (2023): 80. 36721171 10.1186/s13018-022-03467-w PMC9890888

